# Macrophage MSR1 promotes the formation of foamy macrophage and neuronal apoptosis after spinal cord injury

**DOI:** 10.1186/s12974-020-01735-2

**Published:** 2020-02-17

**Authors:** Fan-Qi Kong, Shu-Jie Zhao, Peng Sun, Hao Liu, Jian Jie, Tao Xu, An-Di Xu, Ya-Qing Yang, Ye Zhu, Jian Chen, Zheng Zhou, Ding-Fei Qian, Chang-Jiang Gu, Qi Chen, Guo-Yong Yin, Han-Wen Zhang, Jin Fan

**Affiliations:** 1 Department of Orthopedics, The First Affiliated Hospital of Nanjing Medical University, Nanjing, 210000 Jiangsu China; 2 Department of Orthopedics, The Affiliated Huaian No.1 People’s Hospital of Nanjing Medical University, Huaian, 223001 Jiangsu China; 3 Department of Orthopedics, Pukou Branch of JiangSu Province Hospital (Nanjing Pukou Central Hospital), Nanjing, 211800 China; 4 Key Laboratory of Targeted Intervention of Cardiovascular Disease, Collaborative Innovation Center for Cardiovascular Disease Translational Medicine, Nanjing Medical University, Nanjing, 210000 Jiangsu China

**Keywords:** MSR1, Phagocytosis, Myelin debris, Foamy macrophage, NF-κB

## Abstract

**Background:**

A sustained inflammatory response following spinal cord injury (SCI) contributes to neuronal damage, inhibiting functional recovery. Macrophages, the major participants in the inflammatory response, transform into foamy macrophages after phagocytosing myelin debris, subsequently releasing inflammatory factors and amplifying the secondary injury. Here, we assessed the effect of macrophage scavenger receptor 1 (MSR1) in phagocytosis of myelin debris after SCI and explained its possible mechanism.

**Methods:**

The SCI model was employed to determine the critical role of MSR1 in phagocytosis of myelin debris in vivo. The potential functions and mechanisms of MSR1 were explored using qPCR, western blotting, and immunofluorescence after treating macrophages and RAW264.7 with myelin debris in vitro.

**Results:**

In this study, we found improved recovery from traumatic SCI in MSR1-knockout mice over that in MSR1 wild-type mice. Furthermore, MSR1 promoted the phagocytosis of myelin debris and the formation of foamy macrophage, leading to pro-inflammatory polarization in vitro and in vivo*.* Mechanistically, in the presence of myelin debris, MSR1-mediated NF-κB signaling pathway contributed to the release of inflammatory mediators and subsequently the apoptosis of neurons.

**Conclusions:**

Our study elucidates a previously unrecognized role of MSR1 in the pathophysiology of SCI and suggests that its inhibition may be a new treatment strategy for this traumatic condition.

## Background

Traumatic spinal cord injury (SCI) is a prominent health problem occurrence that brings with tremendous social and economic burdens [1, 2]. The initial mechanical trauma usually triggers a secondary damage cascade, where a sustained inflammatory response to the secondary injury could contribute to neuronal apoptosis, demyelination, and formation of glial scars [3, 4]. Since the initial mechanical trauma is uncontrollable, the treatment of SCI is mainly focused on preventing the secondary damage [5–7].

As the main immune cells, macrophages are recruited to the lesion epicenter at 2–3 days after the SCI and play important roles in the secondary injury [8]. Macrophages are sensitive to microenvironmental signals, in response to which they undergo either pro-inflammatory or anti-inflammatory polarization [9, 10]. In the mouse model of SCI, macrophages are predominantly pro-inflammatory [11, 12]. Zhu et al. further indicated that the transcription profile of macrophages most resembles that of foam cells that reside in atherosclerotic plaques at day 7 post injury [13]. The myelin debris resulting from continuous demyelination contributes to the formation of foamy macrophages, which release inflammatory factors and enhance neurotoxicity [8]. However, the molecular mechanism underlying the formation and function of foamy macrophages remains unclear. Elucidation of the reason why myelin debris promotes largely pro-inflammatory polarization is critical to the development of therapeutic regimens that can reduce pro-inflammatory polarization and enhance protective anti-inflammatory polarization to improve the recovery of patients with SCI.

Macrophage scavenger receptor 1 (MSR1), a member of the scavenger receptor family, participates in many pathophysiological events, such as host defense, endotoxemia, endocytosis, and bone metabolism [14]. Numerous studies have shown that MSR1 is the main mediator of oxidized lipoprotein uptake and promotes the formation of foam cells in atherosclerosis [15, 16]. Reichert et al. also reported that MSR1 promoted the phagocytosis of myelin debris of peripheral nerves in vitro [17]. However, the role of MSR1 in SCI and its impact on the formation and function of foamy macrophages after the primary injury remain poorly understood.

## Materials and methods

### Cell culture and reagents

RAW264.7 cells were obtained from the Cell Bank of the Chinese Academy of Sciences (Shanghai, China). To acquire primary bone marrow-derived macrophages, bone marrow cells were isolated and resuspended in complete RPMI-1640 medium containing 25-ng/ml macrophage colony-stimulating factor (416-ML, R&D Systems, Minneapolis, MN, USA) and then cultured for 7 days. Primary neurons were isolated from embryonic mice according to the instructions of the manual for the Primary Neuron Isolation Kit (88280, Thermo Fisher Scientific, MA, USA). All these cells were cultured according to standard instructions. To test the cytotoxic properties of the foamy macrophages, macrophage and RAW264.7 cell were stimulated with 100 μg/ml myelin debris for 24 h followed by thorough washing with PBS for 3 times and incubation with fresh medium for another 12 h; primary neurons were treated for 3 days with conditioned media obtained from cultures of macrophages and RAW264.7 cells. The antibodies for western blotting in our study included anti-β-actin (Abways, Mouse, Monoclonal, AB0011), anti-MSR1 (Abcam, Rabbit, Polyclonal, ab123946), anti-cleaved-Caspase-3 (CST, Rabbit, Polyclonal, 9662), anti-Bcl2 (CST, Mouse, Monoclonal, 15071), anti-Bax (CST, Rabbit, Monoclonal, 14796), anti-IκBα (CST, Mouse, Monoclonal, 4814), and anti-pIκBα (CST, Rabbit, Monoclonal, 2859). Secondary antibodies for western blotting were purchased from Jackson ImmunoResearch (West Grove, PA, USA). The antibodies for IF in our study were anti-iNOS (Abcam, Rabbit, Polyclonal, ab15323), anti-iNOS (Abcam, Mouse, Monoclonal, ab49999), anti-CD206 (Abcam, Rabbit, Polyclonal, ab64693), anti-F4/80 (Thermo, Mouse, Monoclonal, 14-4801-82), and anti-p65 (CST, Rabbit, Monoclonal, 8242), and secondary antibodies for IF were donkey anti-mouse Alexa Fluor 488 (Abcam, Donkey, Polyclonal, ab150105), goat anti-rabbit Alexa Fluor 594 (Abcam, Goat, Polyclonal, ab150088), and goat anti-rabbit Alexa Fluor 647 (Abcam, Goat, Polyclonal, ab150083). The antibodies for flow cytometry contained F4/80-PE (565410, BD, New York, USA), iNOS-FITC (610330, BD), and CD206-APC (17–2061-82, Thermo). The inhibitors in our study were JSH-23 (S7351, Select, Houston, USA). The IL-1β and TNF-α ELISA kits were obtained from R&D Systems (MLB00C and MTA00B).

### Spinal cord injury

MSR1 KO mice (C57BL/6 background) were generated according to a previously described method, and MSR1 WT mice with identical genetic backgrounds were used as the controls [18]. Genotyping was confirmed via PCR of DNA samples from tail chips (Additional file 5: Figure S4b). The animal protocols were approved by the Animal Committee of the First Affiliated Hospital of Nanjing Medical University. C57BL/6 mice (8–10 weeks old) were used to generate the SCI model. After anesthetizing the animal, a laminectomy was used to expose the spinal cord at T10, and a spinal cord impactor (68097, RWD, CA, USA) was used to create injuries by dropping a rod (weighing 5 g) onto the spinal cord from a height of 6.5 cm. A laminectomy (sham-operated) group without SCI damage was used as the control. Asymmetric SCI was excluded from the experimental analysis.

### Basso Mouse Scale (BMS) behavioral analysis

The motor function of mice was assessed with BMS after injury; each mouse was pretrained and individually placed in an open field [19]. All mice were observed by two independent investigators blinded to the treatment groups on days 1, 3, 7, 14, 21, and 28 after the injury. The BMS scale ranges from 0 (no ankle movement) to 9 (complete functional recovery) and rates locomotion based on hind limb joint movements, trunk position and stability, stepping coordination, paw placement, toe clearance, and tail position.

### Swimming test

A swimming test was used to assess motor function recovery in the mice post SCI. In brief, mice were trained to swim from one end of a water-filled glass tank to the other end. The Louisville Swim Scale was used to evaluate the forelimb dependency, hind limb movement and alternation, trunk instability, and body angle. Each mouse needed to be tested twice, and the final score was based on the mean scores of the two trials.

### Myelin debris isolation and labeling

Sucrose density gradient centrifugation was used to isolate myelin debris from the brains of 8-week-old mice. In brief, the brains were homogenized in 0.32 M sucrose solution and then added on top of a 0.83 M sucrose solution. After centrifugation at 100,000 × *g* for 45 min at 4 °C, the myelin debris was collected from the interface of the two sucrose densities. CFSE (HY-D0938, MCE, NJ, USA) was then used to label the myelin debris through incubation for 30 min at 24 °C.

### Transmission electron microscope (TEM)

As previously reported [20], spinal cord samples were collected at day 7 post injury. Subsequently, after being fixed, dehydrated, stained, and embedded, the samples were sectioned at 70 nm and visualized using a TEM (Tecnai G2 Spirit Bio TWIN, FEI, USA).

### Oil Red O staining

Frozen spinal sections or fixed cells were dried in 100% propylene glycol and then stained with 0.5% Oil Red O solution (G1260, Servicebio, WuHan, China) at 60 °C for 6 min. After processing with 85% propylene glycol for 2 min and rinsing three times with distilled water, the slides were observed under an inverted microscope (Zeiss Scope, Zeiss, Heidenheim, Germany), and the area with Oil Red O stain was quantified with the ImageJ software.

### Histology and immunofluorescence

The mouse hearts were perfused with 0.9% saline followed by 4% paraformaldehyde. The spinal segments surrounding the lesion center were removed and fixed overnight in 4% paraformaldehyde. After dehydration in 15% and 30% sucrose solutions, the samples were frozen and then cut into 10-μm-thick sections for the subsequent experiments. For tissue immunofluorescence staining, the frozen spinal sections were blocked with 10% bovine serum albumin (BSA) and then incubated overnight at 4 °C with the following primary antibodies: anti-NF200 (1:100), anti-GFAP (1:200), anti-F4/80 (1:200), anti-iNOS (1:200), and anti-CD206 (1:200). For cell immunofluorescence staining, the cells were fixed in 4% paraformaldehyde for 30 min, then permeabilized with 0.05% Triton X-100, and finally blocked with 5% BSA. Primary antibodies (anti-iNOS 1:500 and anti-CD206 1:500) were added and the cells were incubated overnight at 4 °C. This was followed by incubation with the following secondary antibodies: donkey anti-mouse Alexa Fluor 488 (1:500), goat anti-rabbit Alexa Fluor 594 (1:500), and goat anti-rabbit Alexa Fluor 647 (1:500). The nuclei were then stained with 4′,6-diamidino-2-phenylindole (DAPI), and microphotographs were obtained using a confocal microscope (LSM710, Zeiss, Heidenheim, Germany). The average intensity of GFAP or NF200 for each field was measured by the ImageJ software.

### Plasmid construction and transfection

The plasmid containing full-length MSR1 and a negative control plasmid were purchased from GenePharma (Shanghai, China). Virus packaging was performed as previously described, and titers were also tested. The cells were infected with 1 × 10^8^ lentivirus transducing units in the presence of 5 μg/mL polybrene (GenePharma, Shanghai, China). After 72 h of culture, infected cells were further selected with 2.5 μg/mL puromycin. Overexpression efficacy of MSR1 was verified by qPCR and western blotting.

### RNA isolation and qPCR

Mice were anesthetized before being sacrificed, and their spinal cord were collected and homogenized with 10 to 15 strokes (3–4 s/stroke) using a homogenizer and plastic pestle on ice. Total RNA of cells and spinal cord were extracted using the Trizol reagent (Takara, Dalian, China) and subsequently converted to cDNA using a reverse transcription kit HiScript II Q RT SuperMix for qPCR (R122-01, Vazyme, China). Next, qPCR reactions were performed using AceQ qPCR SYBR Green Master Mix (Q111-02, Vazyme, China) in a 7500 real-time PCR system (Applied Biosystems, Inc., USA) according to the manufacturer’s instruction. The primer sequences used for qPCR are listed in Additional file 1: Table S1. The mRNA levels of target genes were normalized to the Glyceraldehyde-3-phosphate dehydrogenase (GAPDH) expression. Quantification of qPCR results was performed by the 2 − △CT method.

### Western blotting

Western blotting was carried out as previously described [20]. Proteins were extracted from cells with the protein extraction buffer (Beyotime, Shanghai, China). Spinal cord samples (epicenter ± 0.3 cm) were collected and homogenized with 10 to 15 strokes (3–4 s/stroke) using a homogenizer and plastic pestle with protein extraction buffer. Subsequently, protein concentration was determined using the Bradford method, and equal amounts of proteins were separated by SDS-PAGE gel electrophoresis and transferred onto polyvinylidene fluoride (PVDF) membranes. The membranes were incubated with the following corresponding primary antibodies overnight at 4 °C followed by blocking with 5% skimmed milk or 5% Bovine Serum Albumin (BSA): anti-β-actin (1:1000), anti-MSR1 (1:1000), anti-cleaved-Caspase-3 (1:1000), anti-Bcl2 (1:1000), anti-Bax (1:1000), anti-IκBα (1:1000), and anti-pIκBα (1:1000). After incubating with species-matched secondary antibodies (1:10000), ECL reagents (Share-bio, Shanghai, China) were used to develop bands and the density of protein was accessed by the ImageJ (National Institutes of Health, Bethesda, MD, USA).

### Flow cytometry

To detect the polarization of macrophages, myelin debris-treated macrophages and RAW264.7 cells were collected and then incubated with F4/80-PE, iNOS-FITC, and CD206-APC for 30 min at 4 °C. To analyze the apoptosis of neurons, cells were assessed using an Annexin-V-FITC/PI Apoptosis Detection Kit (556547, BD) according to the manufacturer’s manual. After treatment with conditioned media for 3 days, primary neurons were washed twice with cold PBS. Then, the cells were resuspended in binding buffer that contained 5 μl Annexin-V-FITC and 5 μl propidium iodide (PI) and stained for 10 min at room temperature. Cells were analyzed by flow cytometry (FACSVerse 8, BD Biosciences, Piscataway, NJ, USA) and data analysis was performed using the FlowJo software (Version 7.6.1, Treestar, Ashland, OR, USA).

### ELISA

ELISA was employed to assess the secretion of IL-1β and TNF-α from the supernatants of macrophages. Based on the manufacturer’s instructions, the absorbance was determined using a microplate reader (BioTek, Friedrichshall, Germany) at 450 nm.

### Nissl staining

Frozen spinal sections were treated with Nissl staining solution (G1430, Servicebio) for 1 h at 56 °C and then rapidly dehydrated with absolute ethanol. The sections were finally observed with an inverted microscope (Zeiss Scope, Zeiss, Heidenheim, Germany).

### TUNEL assay

Apoptotic cells were detected with the TUNEL Apoptosis Assay Kit (T2190-50 T, Servicebio) according to the manufacturer’s instructions. The nuclei were stained with DAPI, and images were acquired under a confocal microscope (LSM710, Zeiss, Heidenheim, Germany).

### Statistical analyses

Data are shown as mean ± SD and contain at least three independent biological replicates. One-way analysis of variance (ANOVA) was used for analysis if comparisons were more than two groups, and unpaired 2-tailed Student’s *t* tests were used for two-group comparisons. Differences between groups were considered statistically significant when *p* value < 0.05.

## Results

### MSR1 deficiency improved functional recovery and reduced cellular damage after spinal cord injury

**Fig. 1:**
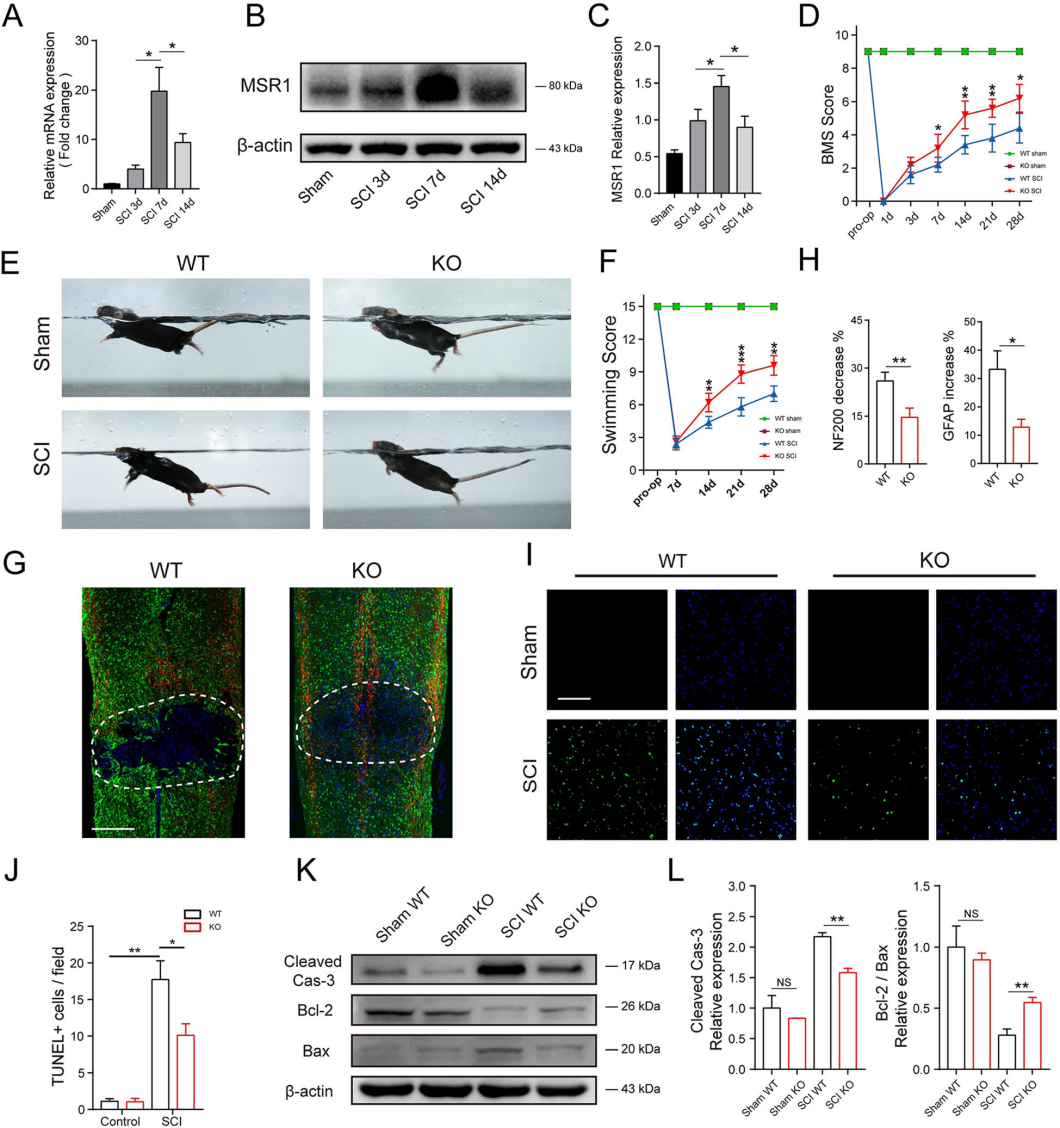
MSR1 deficiency reduced spinal cord damage and motor function deficits after spinal cord injury. **a**–**c** Quantitative PCR and immunoblotting of MSR1 at days 3, 7, and 14 after SCI or sham surgery (*n* = 3 mice per group at each time point, values are the mean ± SD, *p < 0.05, one-way ANOVA). **d** Statistical analysis of the Basso Mouse Scale (BMS) in the MSR1 WT and KO groups over a 28-day period (*n* = 5 mice per group at each time point, values are the mean ± SD, *p < 0.05, **p < 0.01, one-way ANOVA). **e**–**f** Photographs of the various degrees of trunk instability (TI) that were observed after SCI or sham surgery, and statistical analysis of the Louisville Swim Scale in the MSR1 WT and KO groups over a 28-day period (*n* = 5 mice per group at each time point, values are the mean ± SD, **p < 0.01, ***p < 0.001, one-way ANOVA). **g** Representative images of GFAP (in green) and NF200 (in red) in the lesion sites of the MSR1 WT and KO groups at day 28 post injury, nuclei were counterstained with DAPI (blue); the dashed lines indicate the boundary of the damaged area (*n* = 5 per group, scale bar = 500 μm). **h** Quantification of the NF200 intensity increase and GFAP intensity decrease compared with the levels in the sham group (*n* = 5 per group, values are the mean ± SD, *p < 0.05, **p < 0.01, two-tailed Student’s *t* tests). **i**–**j** Representative images showing TUNEL and DAPI co-staining of spinal cord sections of the MSR1 KO and WT mice that were subjected to a sham operation or SCI (*n* = 5 per group, values are the mean ± SD, NS indicates no significance, *p < 0.05, **p < 0.01, one-way ANOVA, scale bar = 200 μm). **k** Western blots of cleaved caspase-3, Bcl-2, and Bax expression in the spinal cords of the MSR1 WT and KO groups that were subjected to a sham operation or SCI. **l** Quantification of activated caspase-3 protein expression and the ratio of Bcl-2 to Bax in the spinal cords of the MSR1 WT and KO groups that were subjected to a sham operation or SCI (*n* = 3 per group, values are the mean ± SD, NS indicates no significance, **p < 0.01, one-way ANOVA)

To investigate the role of MSR1 in the pathophysiology of SCI, quantitative PCR (qPCR) and western blot analysis were used to confirm its expression pattern in the lesion site at 3, 7, and 14 days post injury. Compared with those in the sham operation group, the mRNA and protein levels of MSR1 were significantly upregulated in the SCI group, with the peak of expression observed on day 7 (Fig. 1a–c).

The Basso Mouse Scale (BMS) and Louisville Swim Scale were further used to assess the functional recovery of the MSR1 wild-type (WT) and MSR1 knockout (KO) mice after SCI [21]. As indicated in Fig. 1d and Additional file 2: Figure S1a, there was no obvious difference in the BMS scores and swimming test between the two mouse groups before SCI. In contrast, the functional score for the MSR1 KO mice was significantly superior to that of the MSR1 WT mice beginning on day 7 after SCI. The Louisville Swim Scale also showed that the MSR1 KO mice exhibited less forelimb dependence, faster hind limb alternation, and a smaller angle between the body and water surface beginning on day 7 post injury (Fig. 1e, f). Neurofilament is a surrogate marker of axonal degeneration in central nervous system (CNS) diseases; moreover, the inhibition of axon regeneration and remyelination due to the formation of glial scars is detrimental to the repair of SCI [22–24]. Thus, the 200 kDa subunit of neurofilament (NF200) and glial fibrillary acidic protein (GFAP) were used to evaluate the neuronal and axonal damage after SCI. The magnitude of NF200 decline and GFAP increase (relative to the levels in the sham group) in the lesion areas of MSR1 KO mice was lower than that of the MSR1 WT group after SCI (Fig. 1g, h and Additional file 2: Figure S1b). The terminal deoxynucleotidyl transferase dUTP nick-end labeling (TUNEL) assay was used to assess apoptotic cells in the lesion core of SCI [25]. As indicated in Fig. 1 i and j, the percentage of TUNEL-positive cells was lower in the MSR1 KO group than in the MSR1 WT group at day 7 post SCI. Western blot analysis also demonstrated that the level of cleaved caspase-3 in the lesion core was considerably lower, whereas the ratio of B-cell lymphoma-2/BCL2-associated X protein (Bcl-2/Bax, a key determinant of apoptosis) was significant higher, in the MSR1 KO mice than in the MSR1 WT mice after SCI (Fig. 1k, l). In summary, MSR1 may aggravate motor dysfunction and cellular damage post SCI.

### Macrophage MSR1 mediated myelin debris phagocytosis after SCI

**Fig. 2:**
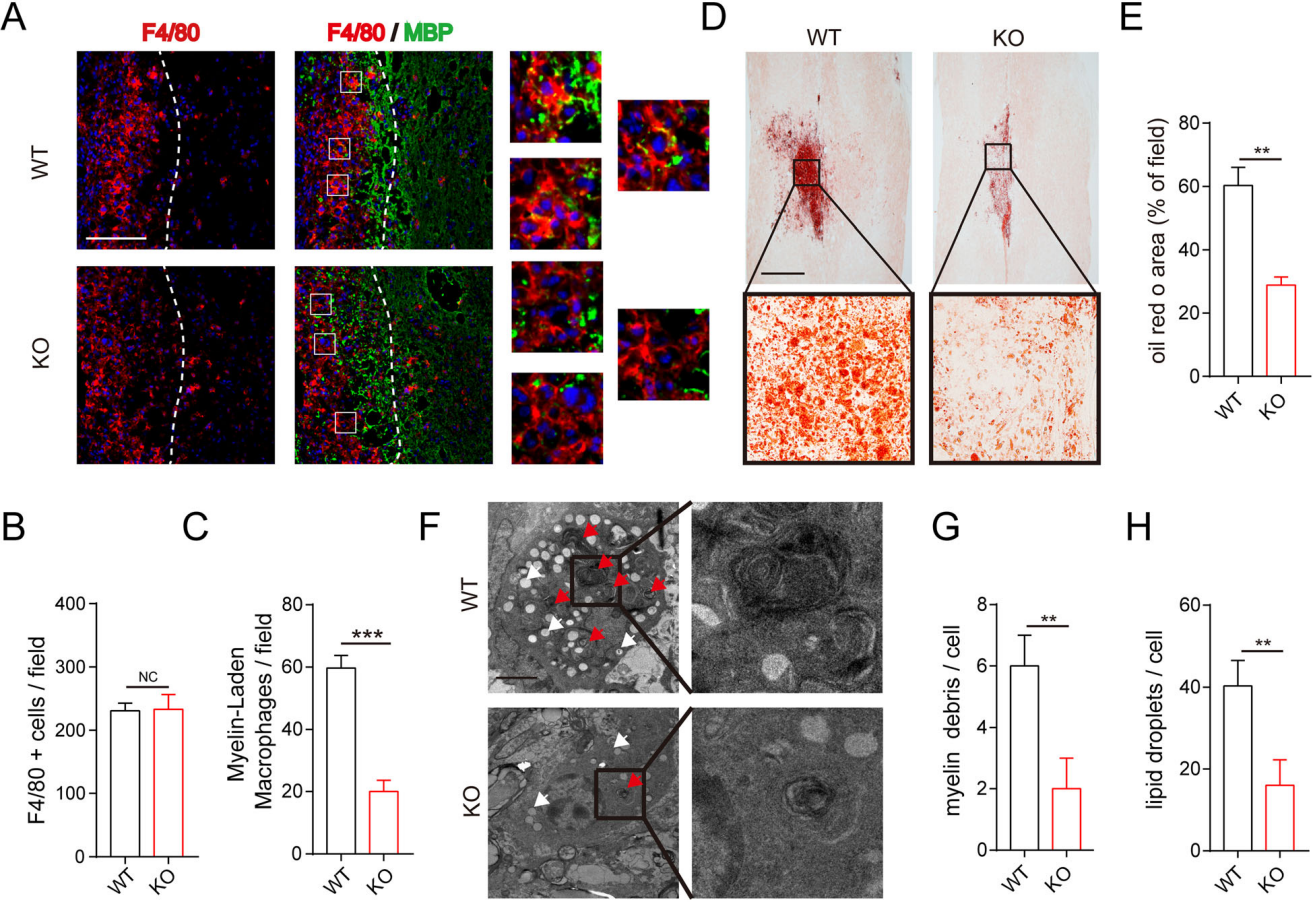
Myelin debris phagocytosis was reduced in MSR1 KO macrophages after spinal cord injury. **a** IF staining of the macrophage marker F4/80 (red) and the myelin marker MBP (green) in the damage areas of the MSR1 WT and KO groups at day 7 post SCI. Nuclei were counterstained with DAPI (blue). Scale bar = 200 μm. **b**–**c** Quantification of F4/80+ macrophages and myelin-laden macrophages in the lesion site at day 7 post injury (*n* = 3 per group, values are the mean ± SD, NS indicates no significance, **p < 0.01, two-tailed Student’s *t* tests). **d** Microscopic images of Oil Red O staining of the lesion sites at day 7 post injury in the MSR1 WT and KO groups. Scale bar = 500 μm. **e** Statistical analysis of the Oil Red O-stained areas in the MSR1 WT and KO groups (*n* = 3 per group, values are the mean ± SD, **p < 0.01, two-tailed Student’s *t* tests). **f** Transmission electron microscopy images of foamy macrophages in the lesion sites of the MSR1 WT and KO mice on day 7 post injury. Red arrows indicate degrading myelin debris, and white arrows indicate lipid droplets. Scale bars = 5 μm. **g**–**h** Numbers of myelin debris and lipid droplets in macrophages of the MSR1 WT mice on day 7 post injury (*n* = 3 per group, values are the means ± SD, **p < 0.01, two-tailed Student’s *t* tests)

Recent reports have shown that macrophages are recruited to the lesion epicenter and play an important role in phagocytosing myelin debris [8, 26]. To investigate whether MSR1 is involved in this process, we assessed F4/80 (a marker of total macrophages) and myelin basic protein (MBP) immunoreactivity in the lesion sites of the MSR1 WT and KO mice at day 7 post SCI. There were no significant differences in macrophage infiltration between the two mouse groups (Fig. 2a, b). However, the macrophages from MSR1 WT mice contained numerous myelin debris puncta; the staining pattern was absent in those of the MSR1 KO mice, suggesting reduced phagocytic activity in the KO group (Fig. 2a, c). After phagocytosis, myelin debris are delivered to the lysosomes and packaged as neutral lipids into intracellular lipid droplets, eventually leading to the formation of foamy macrophages [8]. Oil Red O staining is highly specific for those intracellular neutral lipids [27]. As indicated in Fig. 2 d and e, the lesion core in the MSR1 KO mice showed significantly less Oil Red O staining than that in the MSR1 WT mice at day 7 post injury, suggesting a limitation in the formation of foamy macrophages in the mice lacking MSR1. The foamy macrophages containing myelin debris in the lesion section were further clearly evident by transmission electron microscopy, and the number of lipid vacuoles and myelin debris in macrophages from the MSR1 KO mice were significantly less than those in macrophages from the MSR1 WT mice (Fig. 2f–h). Taken together, the results suggest that the depletion of MSR1 may prevent the formation of foamy macrophages after SCI.

### MSR1 promoted the myelin debris phagocytosis and formation of foamy macrophages in vitro

**Fig. 3:**
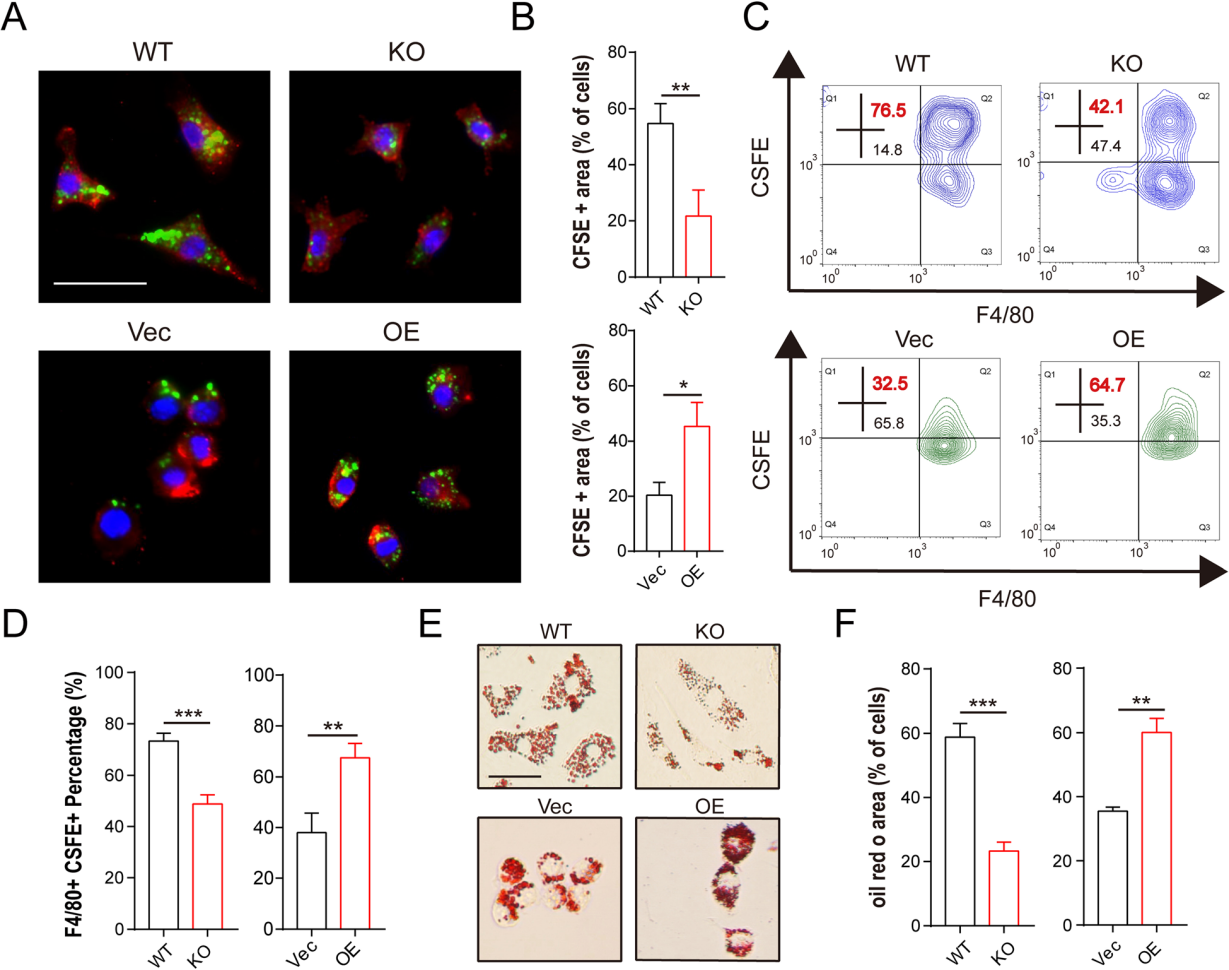
Myelin debris phagocytosis was reduced in MSR1 KO macrophages and increased in MSR1-overexpressed RAW264.7 cells in vitro. **a** Representative immunofluorescence images showing the engulfment of CFSE-labeled myelin debris (green) by different groups of macrophages (F4/80, red) after treatment with myelin debris. Scale bars = 20 μm. **b** Phagocytosis of myelin debris, as determined by counting the percentage of the CFSE+ area (*n* = 3 per group, values are the mean ± SD, **p < 0.01, two-tailed Student’s *t* tests). **c** Flow cytometric analysis of macrophages after treatment with CFSE-labeled myelin debris. The F4/80+ and CFSE+ quadrant represent myelin-laden macrophages. **d** Calculated percentages of F4/80+ and CFSE+ macrophages in different groups (MSR1 WT vs KO, MSR1 Vec vs OE) (*n* = 3 per group, values are the mean ± SD, ***p < 0.001, two-tailed Student’s *t* tests). **e** Oil Red O staining of foamy macrophages after treatment with myelin debris for 24 h. Scale bar = 20 μm. **f** Statistical analysis of the Oil Red O-stained areas in different groups of foamy macrophages (*n* = 3 per group, values are the mean ± SD, **p < 0.01, ***p < 0.001, two-tailed Student’s *t* tests)

To further confirm whether macrophage MSR1 participated in the modulation of myelin debris phagocytosis in vitro, we isolated primary macrophages from MSR1 WT and KO mice and overexpressed MSR1 in the RAW264.7 cell line. The MSR1 KO and overexpression efficiencies were then confirmed by qPCR and western blot assays (Additional file 2: Figure S1c). Then, to track the process of myelin debris engulfment, the pieces of debris were marked with carboxyfluorescein succinimidyl ester (CFSE), a noncytotoxic dye [28]. The primary macrophages and RAW264.7 cells were then incubated with the CFSE-labeled myelin debris, and the internalized pieces were visualized using standard green fluorescent protein filter sets of the fluorescence microscope. As shown in Fig. 3 a and b, primary macrophages from the MSR1 KO mice revealed a smaller CFSE-positive population compared with those from the MSR1 WT mice, and more phagocytosed CFSE-labeled myelin debris was observed in the MSR1-overexpressing RAW264.7 cells than in the control cells. A similar result was reflected by the percentage of cells staining positive for both F4/80 and CFSE, assessed using flow cytometry (Fig. 3c, d and Additional file 3: Figure S2a). Oil Red O staining was used to quantify the intracellular lipid accumulation of the foamy macrophages, with the macrophages from the MSR1 WT mice and MSR1-overexpressing RAW264.7 cells showing more lipid droplets within the cytoplasm (Fig. 3e, f). Collectively, our results suggest that MSR1 contributes to the phagocytosis of myelin debris and formation of foamy macrophages in vitro.

### MSR1 promoted pro-inflammatory polarization of foamy macrophages in vitro and in vivo

**Fig. 4:**
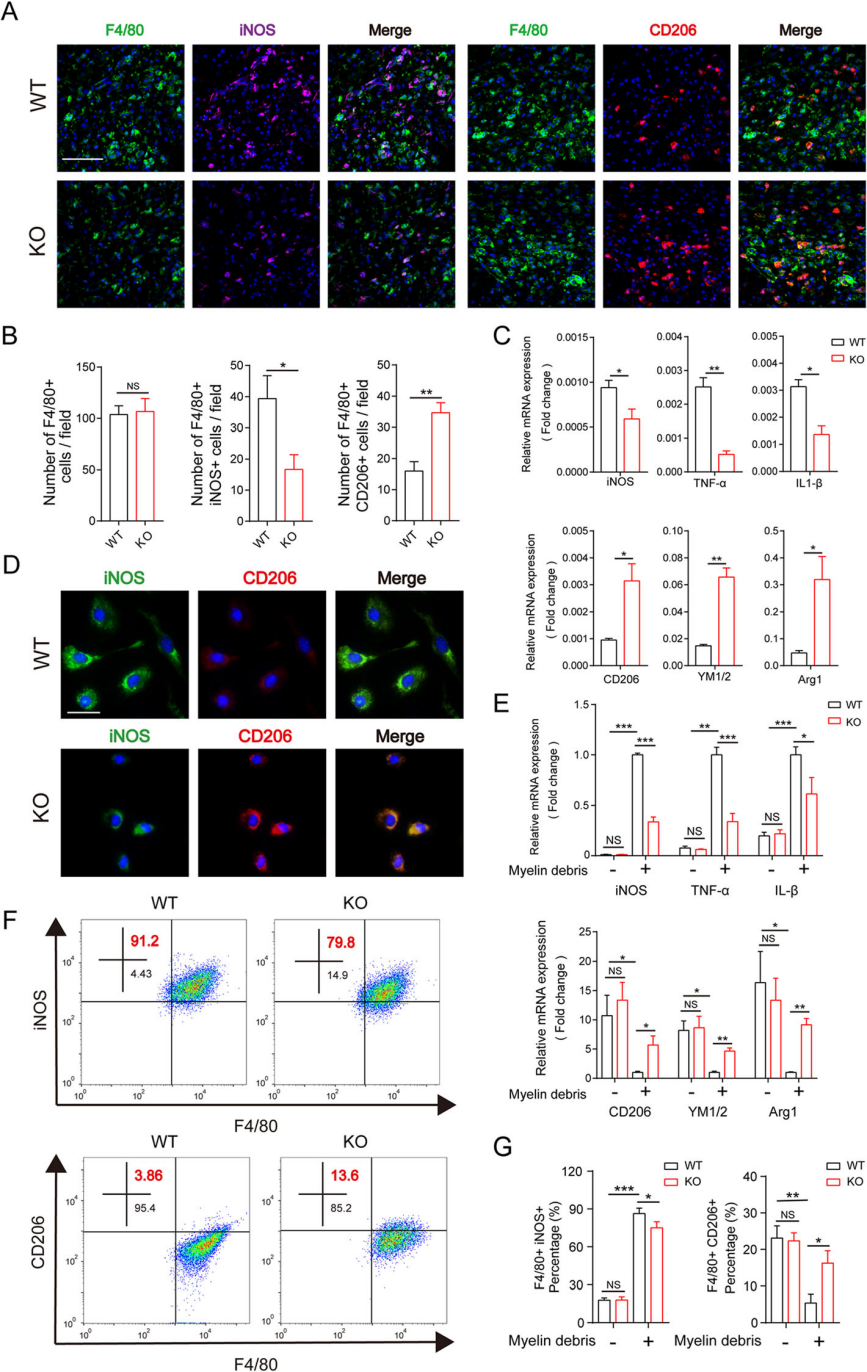
MSR1 knockout inhibited pro-inflammatory polarization of macrophages after treatment with myelin debris in vitro. **a** IF staining of F4/80 (green), iNOS (pro-inflammatory macrophage marker, purple), and CD206 (anti-inflammatory macrophage marker, red) in the lesion sites of the MSR1 WT and KO mice. Bar = 200 μm. **b** Determination of the pro-inflammatory and anti-inflammatory macrophages by analysis the number of iNOS+ and F4/80+ cells or CD206+ and F4/80+ cells in the lesion sites of the MSR1 WT and KO mice (*n* = 3 per group, values are the mean ± SD, NS indicates no significance, *p < 0.05, **p < 0.01, two-tailed Student’s *t* tests). **c** Quantitative PCR of pro-inflammatory macrophage marker genes (iNOS, TNF-α, and IL-β) and anti-inflammatory macrophage marker genes (CD206, YM1/2, and Arg1) in the lesion sites of the MSR1 WT and KO mice at day 7 post injury (*n* = 3 per group, values are the mean ± SD, *p < 0.05, **p < 0.01, ***p < 0.001, one-way ANOVA). **d** IF staining of iNOS and CD206 in MSR1 WT and KO macrophages after treatment with myelin debris for 24 h. Scale bar = 10 μm. **e** Quantitative PCR of pro-inflammatory macrophage marker genes (iNOS, TNF-α, and IL-1β) and anti-inflammatory macrophage marker genes (CD206, YM1/2, and Arg1) in MSR1 WT and KO macrophages before or after treatment with myelin debris (*n* = 3 per group, values are the mean ± SD, NS indicates no significance, *p < 0.05, **p < 0.01, ***p < 0.001, one-way ANOVA). **f**–**g** Flow cytometric analysis of myelin debris-treated macrophages from MSR1 WT or KO mice. The percentages of F4/80+ iNOS+ and F4/80+ CD206+ macrophages in the MSR1 WT and KO groups were calculated (*n* = 3 per group, values are the mean ± SD, NS indicates no significance, *p < 0.05, **p < 0.01, ***p < 0.001, one-way ANOVA)

It is known that macrophage that phagocytoses myelin debris contributes to the inflammatory responses in SCI progression [29, 30]. Therefore, we further explore whether MSR1, which enhances the phagocytic ability of myelin debris, promotes the polarization of pro-inflammatory macrophages. As demonstrated in Fig. 4 a and b, there were no significant differences in number of F4/80-positive cells between the two mouse groups, but a marked decrease in the inducible nitric oxide synthase (iNOS)-positive macrophage fraction and a higher level of arginase-1 (Arg1) in the macrophages were observed in the lesion areas from the MSR1 KO mice at day 7 post SCI. The mRNA expression levels of pro-inflammatory markers (iNOS, TNF-α, and IL-1) were decreased whereas those of anti-inflammatory markers (CD206, YM1/2, and Arg1) were increased in the lesion areas of MSR1 KO mice relative to the levels in the MSR1 WT mice (Fig. 4c).

Next, we isolated the primary macrophages from the MSR1 WT and KO mice and treated them with myelin debris for 24 h and then used immunofluorescence to explore their polarization phenotype. As shown in Fig. 4d, a weaker iNOS (green) fluorescence intensity and a stronger CD206 (red) fluorescence intensity were observed in the MSR1 KO macrophages. The qPCR results also revealed that the pro-inflammatory markers (iNOS, TNF-α, and IL-1β) were decreased, and the anti-inflammatory markers (CD206, YM1/2, and Arg1) were increased in the MSR1 KO macrophages (Fig. 4e). Similar results were observed in the flow cytometric analysis, where the ratio of anti-inflammatory macrophages in F4/80-positive cells was higher and the percentage of F4/80-positive and iNOS-positive cells was lower in the MSR1 KO group (Fig. 4f, g). It was notable that no statistical differences in the groups without myelin debris (Fig. 4e, g and Additional file 4: Figure S3a and b).

**Fig. 5:**
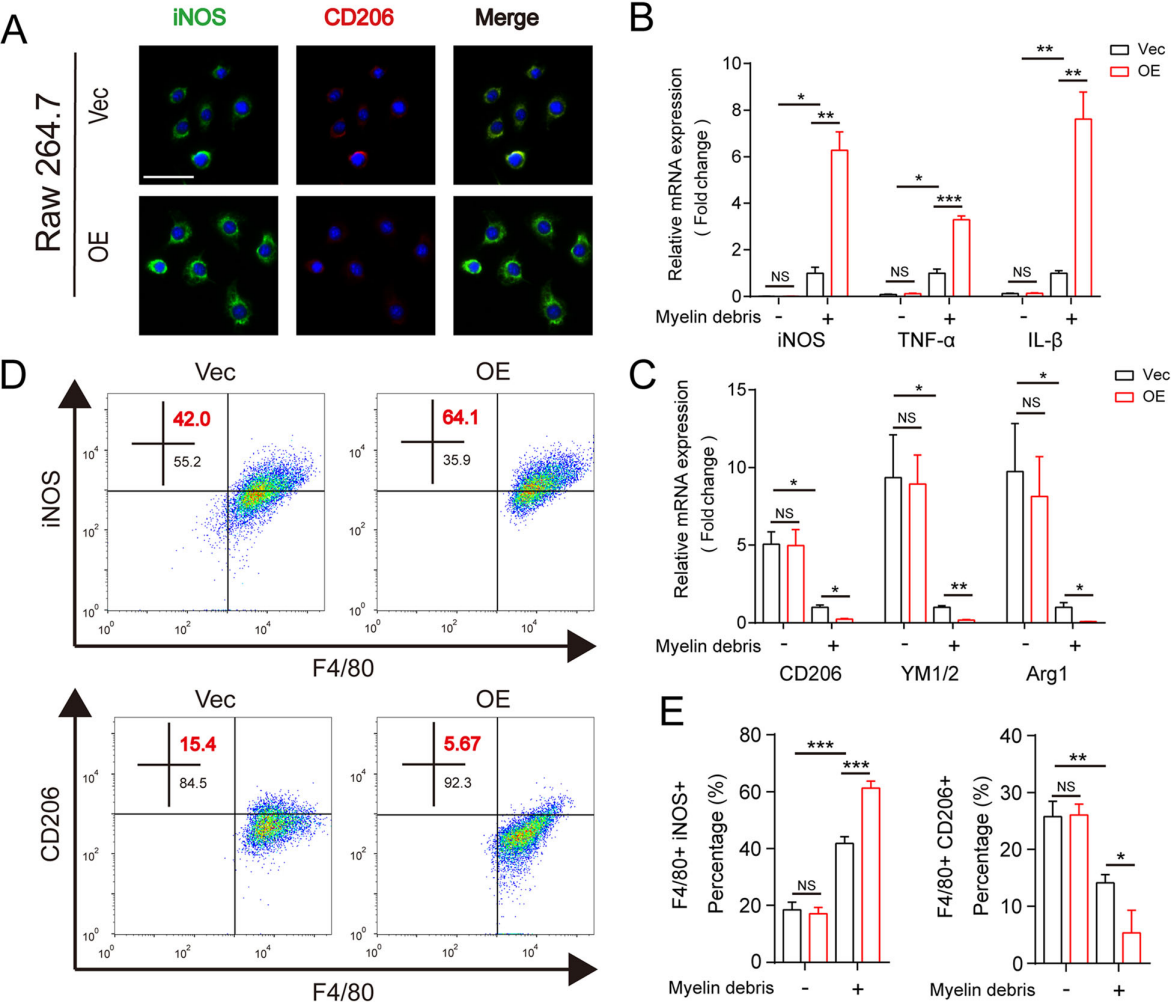
MSR1 overexpression promoted the pro-inflammatory polarization of RAW264.7 cells after treatment with myelin debris in vitro. **a** IF staining of iNOS and CD206 in MSR1 vector (Vec) and overexpression (OE) RAW264.7 cells after treatment with myelin debris for 24 h. Scale bar = 10 μm. **b**, **c** Quantitative PCR of pro-inflammatory macrophage marker genes (iNOS, TNF-α, and IL-1β) and anti-inflammatory macrophage marker genes (CD206, YM1/2, and Arg1) in MSR1 Vec and OE RAW264.7 cells before or after treatment with myelin debris for 24 h (*n* = 3 per group, values are the mean ± SD, NS indicates no significance, *p < 0.05, **p < 0.01, ***p < 0.001, one-way ANOVA). **d**, **e** Flow cytometric analysis of MSR1 Vec and OE RAW264.7 cells after treatment with myelin debris for 24 h. The percentages of F4/80+ iNOS+ and F4/80+ CD206+ macrophages in the different groups were calculated (*n* = 3 per group, values are the mean ± SD, *p < 0.05, ***p < 0.001, one-way ANOVA)

To further verify the reliability of the results, we analyzed the alteration of the polarization phenotype in MSR1-overexpressing RAW264.7 cells after their treatment with myelin debris. As shown in Fig. 5 a–e, the results of fluorescence staining, qPCR, and flow cytometric analysis all demonstrated that MSR1-overexpressed RAW264.7 cells promoted the pro-inflammatory phenotype after the treatment with myelin debris. However, the polarization of RAW264.7 did not change between different groups in the absence of myelin debris (Additional file 4: Figure S3a and b). Taken together, our data indicate that MSR1 may affect the polarization of macrophages and RAW264.7 cells after treatment with myelin debris.

### Foamy macrophage MSR1 promoted the release of pro-inflammatory cytokines via NF-κB signaling pathway

**Fig. 6:**
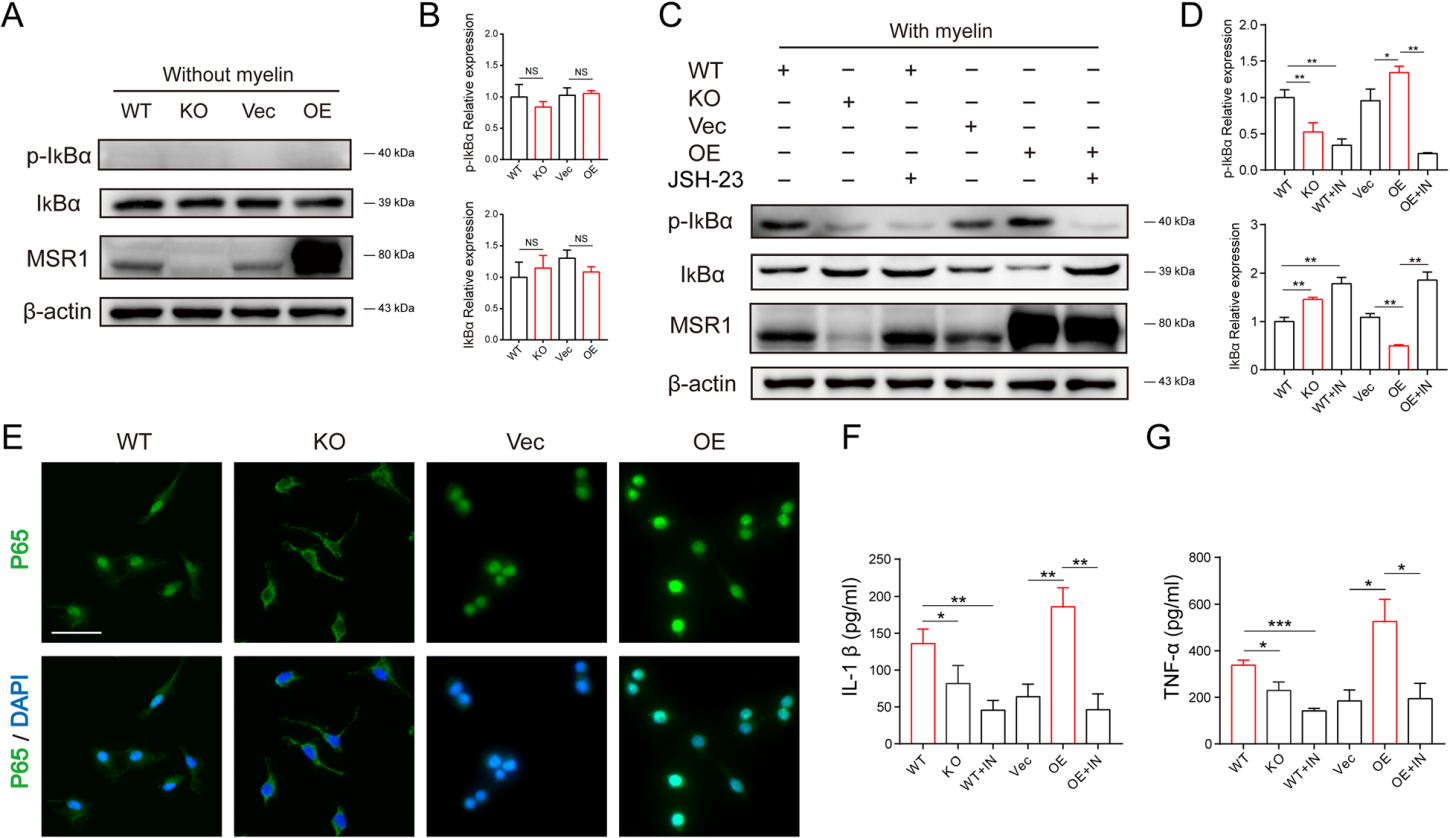
MSR1 mediated activation of the NF-κB signaling pathway and the release of inflammatory factors after treatment with myelin debris. **a**, **b** Immunoblot images showing the effect of MSR1 knockout or overexpression on the expression of p-IκBα/IκBα with absence of myelin debris (*n* = 3 per group, values are the mean, NS indicates no significance, one-way ANOVA). **c**, **d** Altered protein expression level of p-IκBα/IκBα was detected using western blotting in MSR1 knockout or overexpression groups after treatment with myelin debris, or MSR1 WT macrophages and MSR1-overexpressed RAW264.7 cells treated with JSH-23 (an inhibitor of NF-κB), before treatment with myelin debris (*n* = 3 per group, values are the mean ± SD, *p < 0.05, **p < 0.01, one-way ANOVA, IN = JSH-23). **e** IF staining was used to analyze the distribution of p65 (green) in MSR1 WT and KO macrophages, and MSR1 Vec and OE RAW264.7 cells. The cell nuclei were stained with DAPI (blue fluorescence), scale bars = 20 μm. **f**–**g** MSR1 WT and KO macrophages and MSR1 Vec and OE RAW264.7 cells were treated with myelin debris for 24 h, or MSR1 WT macrophages and MSR1 OE RAW264.7 cells treated with JSH-23 before adding myelin debris, and amount of secreted IL1-β and TNF-α in the supernatants of cell culture were detected by ELISA (*n* = 3 per group, values are the mean ± SD, *p < 0.05, **p < 0.01, ***p < 0.001, one-way ANOVA, IN = JSH-23)

The NF-κB signaling pathway is a key participant in macrophage polarization and the inflammatory response [31, 32]. Also, some reports have pointed out that MSR1 was involved in the activation of the NF-κB signaling pathway [33]. Therefore, we examined whether MSR1 regulates NF-κB signaling pathway in macrophages after their treatment with myelin debris. In the absence of myelin debris, MSR1 did not affect the phosphorylation and degradation of IκBα (an inhibitor of NF-κB transcriptional activity) (Fig. 6a, b). As indicated in Fig. 6 c and d, MSR1 KO decreased the level of phosphorylated IκBα, whereas MSR1 overexpression positively influenced the phosphorylation of IκBα in the presence of myelin debris. IF was also used to detect the subcellular distribution of p65, a major component of NF-κB. After treatment with myelin debris for 2 h, less nuclear-localized p65 was detected in the macrophages from the MSR1 KO mice, whereas more p65 moved into the nuclei in the MSR1-overexpressing RAW264.7 cells relative to that in the control cells (Fig. 6e). The injured environment during the secondary damage cascade is dominated by the presence of the pro-inflammatory cytokine tumor necrosis factor α (TNF-α); interleukin-1β(IL-1β) and NF-κB signaling pathway are associated with the release of those inflammatory factors [3]. ELISA was used to detect the content of inflammatory factors in the conditioned media from cultures of macrophages and RAW264.7 cells treated with myelin debris. As indicated in Fig. 6 f and g, the protein levels of IL-1β and TNF-α were lower in the conditioned medium of the macrophages from MSR1 WT mice. Additionally, the contents of IL-1β and TNF-α were obviously increased in the conditioned medium of the MSR1-overexpressing RAW264.7 cells. To further confirm that the NF-κB signaling pathway was involved in the MSR1-mediated release of inflammatory factors, small molecule inhibitor targeting this signaling pathway (JSH-23) was used. With the inhibition of IκBα phosphorylation (Fig. 6c, d), the levels of IL-1β and TNF-α were also significantly reduced in the MSR1 WT mouse-derived macrophages and MSR1-overexpressing RAW264.7 cells (Fig. 6f, g). These results suggest that MSR1 mediates activation of the NF-κB signaling pathway and the subsequent release of inflammatory factors in macrophages and RAW264.7 cells in the presence of myelin debris.

### Effects of foamy macrophage MSR1 on neuronal apoptosis

**Fig. 7:**
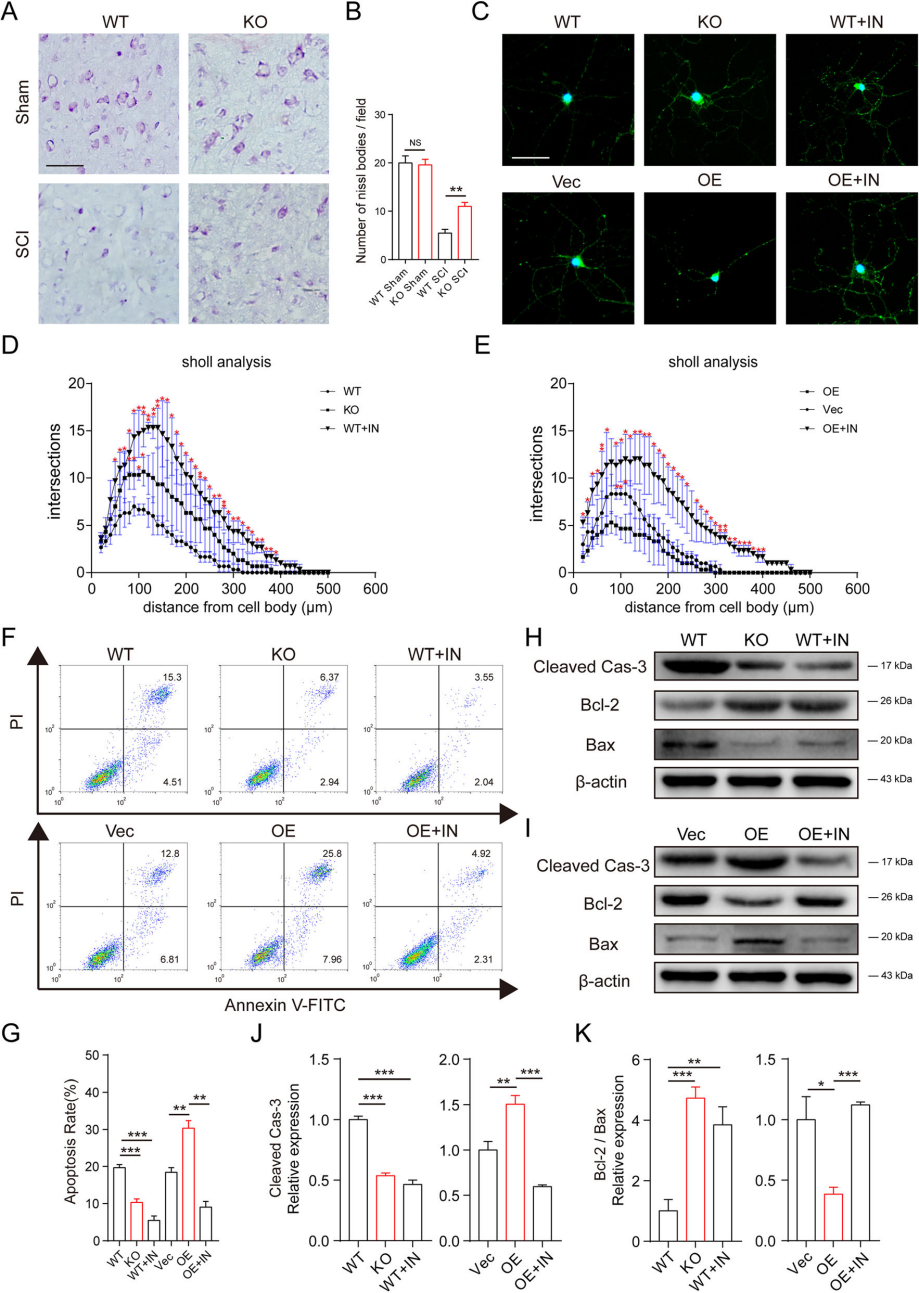
MSR1 promoted foamy macrophage-mediated neuronal apoptosis. **a**, **b** Representative photomicrographs of the Nissl-stained neurons in MSR1 WT and KO groups after SCI or sham surgery and the number of Nissl bodies was calculated (*n* = 3 per group, values are the mean ± SD, NS indicates no significance, **p < 0.01, one-way ANOVA, scale bars = 200 μm). **c** Representative images of neurons in neuronal cultures treated with the conditioned medium from different groups of macrophages and RAW 264.7 cells (*n* = 3 per group, MSR1 WT, MSR1 KO, MSR1 OE, MSR1 Vec, MSR1 WT + JSH-23, MSR1 OE + JSH-23) in the presence of myelin debris. Scale bar = 100 μm. **d**–**e** Sholl analysis of neurite growth in different groups (*n* = 3 per group, values are the mean ± SD, *p < 0.05, **p < 0.01, one-way ANOVA, IN = JSH-23). **f**, **g** Flow cytometric analysis of Neuronal apoptosis in different groups through Annexin V-FITC/PI double staining (*n* = 3 per group, values are the mean ± SD, **p < 0.01, ***p < 0.001, one-way ANOVA). **h**, **i** Western blot analysis of apoptosis-related proteins in neurons cultured in the conditioned media obtained from different groups. **j**, **k** Quantification of activated caspase-3 protein expression and the ratio of Bcl-2 to Bax in primary neurons cultured with the conditioned media obtained from different groups. Beta-actin was used as loading control (*n* = 3 per group, values are the mean ± SD, *p < 0.05, **p < 0.01, ***p < 0.001, one-way ANOVA)

To investigate the effect of foamy macrophage MSR1 on neurons, Nissl staining was used to observe the morphology and number of neurons in the MSR1 WT and KO groups. As indicated in Fig. 7 a and b, there was no significant difference in Nissl-positive neurons between the MSR1 KO and MSR1 WT groups before injury. However, in the MSR1 WT mice post injury, the Nissl-positive neurons were more reduced in quantity and had obvious shrunken cell bodies and pyknotic nuclei (Fig. 7a, b). To further illustrate the effects of the inflammatory factors secreted by foamy macrophages on neurons in vitro, primary neurons and macrophages were isolated and western blot analysis revealed that MSR1 was expressed only on the macrophages, excluding the influence of the neuronal MSR1 deletion (Additional file 5: Figure S4a). The primary neurons were treated for 3 days with conditioned media obtained from foamy macrophages (WT vs KO) and RAW264.7 cells (Vec vs OE) treated with myelin debris. Sholl analysis revealed that myelin debris-treated MSR1 WT macrophages and MSR1-overexpressing RAW264.7 cell supernatants reduced the length of neurite growth, as well as the total number of neuritic branches, as compared with supernatants of MSR1 KO and Vec groups (Fig. 7c–e). Furthermore, NF-κB signaling pathway inhibitor (JSH-23) reduced those influences on neurons (Fig. 7c–e). Flow cytometry showed that neuronal apoptosis was significantly increased in the conditioned media from the MSR1 WT macrophages and MSR1-overexpressing RAW264.7 cells compared with the corresponding control groups (Fig. 7f, g and Additional file 3: Figure S2b). Similar results were obtained by western blot analysis, where the expression of activated caspase-3 protein was higher in primary neurons treated with conditioned media from MSR1 WT macrophages and MSR1-overexpressing RAW264.7 cells, and the ratio of Bcl-2/Bax was lower than that in primary neurons treated with conditioned media from MSR1 KO macrophages and control vector-carrying RAW264.7 cells (Fig. 7h–k). Adding JSH-23 in advance could alleviate the effect of macrophage and RAW264.7 cell MSR1 on neuronal apoptosis (Fig. 7f–k). Taken together, the results suggest that MSR1 promoted foamy macrophage mediation of neuronal apoptosis through NF-κB signaling pathway.

## Discussion

As a scavenger receptor on macrophages, MSR1 has been reported to play several roles in the host defense against pathogenic bacteria [14]. There are also many published studies that have demonstrated that MSR1 contributes to the pathophysiology of brain stroke, cardiac infarction, and bone metabolism [14]. However, seldom literatures report the role of MSR1 in SCI. In this present study, we found that MSR1 promoted the formation of foamy macrophages and mediated the detrimental effects of SCI. Furthermore, macrophage MSR1 promoted the release of inflammatory cytokines by activating the NF-κB signaling pathway, leading to apoptosis of the neurons.

In the central nervous system (CNS), the clearance of myelin debris is executed mainly through phagocytosis by macrophages [8]. Recent studies have revealed that microglial cells are distributed mainly at the margin of injury and at undamaged areas and that their efficiency in myelin debris phagocytosis is much lower than that of macrophages [8, 34]. Therefore, our study mainly focused on investigating the relationship between the macrophage MSR1 and myelin debris in SCI. In different disease models, the effect of myelin debris on macrophage polarization in vivo remains controversial [8, 35, 36]. Myelin-laden macrophages express multiple anti-inflammatory molecules in multiple sclerosis and arthrosclerosis [35, 36]. In contrast, the formation of foamy macrophages is associated with the upregulation of pro-inflammatory polarization in SCI [8]. Meanwhile, there are also conflicting results from the stimulation of macrophages with myelin debris in vitro. Some literatures reported that myelin phagocytosis induce release of inflammatory factors [29, 37, 38], while others showed that myelin-laden macrophages express anti-inflammatory mediators in response to pro-inflammatory stimuli, and the molecular mechanism that gives rise to such contradictory findings is still not clear [30, 35, 39]. In our study, myelin debris contributed to the formation of foamy macrophages and led to the pro-inflammatory polarization of macrophages after SCI.

The NF-kB signaling pathway is widely considered to be associated with the transcription of pro-inflammatory cytokines and the polarization of pro-inflammatory macrophages [40]. Yu et al. showed that MSR1 was required for the lipopolysaccharide-induced activation of the NF-κB signaling pathway in macrophages [33]. Moreover, recent evidence also suggests that myelin debris can activate the FAK/Akt/NF-κB pathways in macrophages [29]. In the present study, we discovered that MSR1 mediated the activation of the NF-κB signaling pathway in macrophages after their treatment with myelin debris and led to the release of inflammatory factors. Thus, manipulation of the inflammatory microenvironment through the targeting of MSR1 may serve as a new therapeutic approach for SCI.

In our study, the SCI model was created using MSR1 KO mice, and whether the change in foamy macrophages in vivo was caused by the indirect effect of other cells during SCI could not be fully determined; thus, further experiments using conditional KO mice with a higher specificity for macrophages are required to verify this. Moreover, because the phagocytosis of myelin debris by macrophages is closely related to lipid metabolism, further experiments are also required to verify whether MSR1 affects myelin lipid metabolism and lipid efflux [41]. In addition, we investigated that macrophage MSR1 promoted the phagocytosis of myelin debris and release of pro-inflammatory cytokines via NF-κB signaling pathway. We cannot, however, rule out the possibility that MSR1 only regulates phagocytosis of the macrophages, and subsequent effects are caused by engulfed myelin debris.

## Conclusions

This study revealed that MSR1 aggravates SCI by promoting the formation of foamy macrophages and inducing pro-inflammatory polarization. This effect correlated with activation of the NF-kB signaling pathway and the release of inflammatory factors, ultimately leading to neuronal apoptosis. Our data elucidates a previously unrecognized role of MSR1 in the pathophysiology of SCI, and further enriched the understanding of the relationship between myelin debris and macrophages in neuroimmune.

## Supplementary information

**Additional file 1 **: **Table S1.** The sequences of primers.

**Additional file 2 **: **Figure S1.** (a) Photographs of swimming tests of the MSR1 WT and KO mice before injury, *n*=5 mice per group. (b) IF staining of GFAP (in green) and NF200 (in red) in the spinal cord of the MSR1 WT and KO mice before injury, nuclei were counterstained with DAPI (blue). Scale bar = 500 μm. (c) The knockout and overexpression efficiency of MSR1 in macrophages and RAW264.7 cells were also confirmed by qPCR and western blotting (*n* = 3 per group, values are the mean ± SD, **p < 0.01, one-way ANOVA).

**Additional file 3 **: **Figure S2.** (a) Flow cytometry plots showing the gating strategy to identify macrophages and RAW264.7 cells. SSC-A = side scatter-area; FSC-A = forward scatter-area. (b) Flow cytometry plots showing the gating strategy to identify neurons. SSC-A = side scatter-area; FSC-A = forward scatter-area.

**Additional file 4 **: **Figure S3.** (a) IF staining of iNOS, CD206, and F4/80 in different groups of macrophages and RAW264.7 cells (WT vs KO, Vec vs OE) in absence of myelin debris, *n*=3 per group. Scale bar = 20 μm. (b) Flow cytometric analysis of iNOS, CD206, and F4/80 in different groups of macrophages and RAW264.7 cells (WT vs KO, Vec vs OE) in absence of myelin debris, *n*=3 per group.

**Additional file 5 **: **Figure S4.** (a) Immunoblot images showing the expression patterns of MSR1 in macrophages and neurons, *n*=3 per group. (b) The genotyping of MSR1 WT or MSR1 KO mice was confirmed by PCR of DNA samples from tail chips.

## Data Availability

The datasets used and/or analyzed during the current study are available from the corresponding author on reasonable request.

## Abbreviations

Arg1: Arginase-1
Bcl-2/Bax: B-cell lymphoma-2/BCL2-associated X protein
BMS: The Basso Mouse Scale
CFSE: Carboxyfluorescein succinimidyl ester
CNS: Central nervous system
GFAP: Glial fibrillary acidic protein
IL-1β: Interleukin-1β
iNOS: Inducible nitric oxide synthase
KO: Knockout
MBP: Myelin basic protein
MSR1: Macrophage scavenger receptor 1
NF200: The 200 kDa subunit of neurofilament
qPCR: Quantitative PCR
SCI: Spinal cord injury
TEM: Transmission electron microscope
TNF-α: Tumor necrosis factor α
TUNEL: The terminal deoxynucleotidyl transferase dUTP nick-end labeling
WT: Wild-type
