# Supplementary figures and images for: Macrophage MSR1 promotes the formation of foamy macrophage and neuronal apoptosis after spinal cord injury
Source: J Neuroinflammation. 2020 Feb 17;17:62. doi: 10.1186/s12974-020-01735-2 (PMC7027125; doi:10.1186/s12974-020-01735-2)

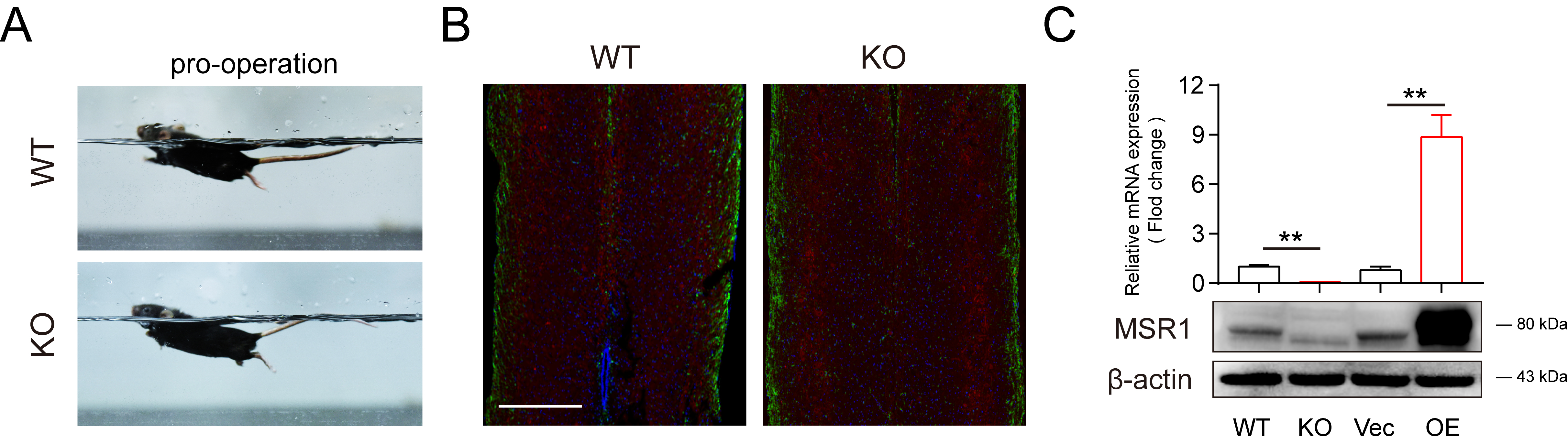

Supplement: Supplementary file 2 — Additional file 2 : Figure S1. (a) Photographs of swimming tests of the MSR1 WT and KO mice before injury, n=5 mice per group. (b) IF staining of GFAP (in green) and NF200 (in red) in the spinal cord of the MSR1 WT and KO mice before injury, nuclei were counterstained with DAPI (blue). Scale bar = 500 μm. (c) The knockout and overexpression efficiency of MSR1 in macrophages and RAW264.7 cells were also confirmed by qPCR and western blotting (n = 3 per group, values are the mean ± SD, **p < 0.01, one-way ANOVA). [file 12974_2020_1735_MOESM2_ESM.jpg]

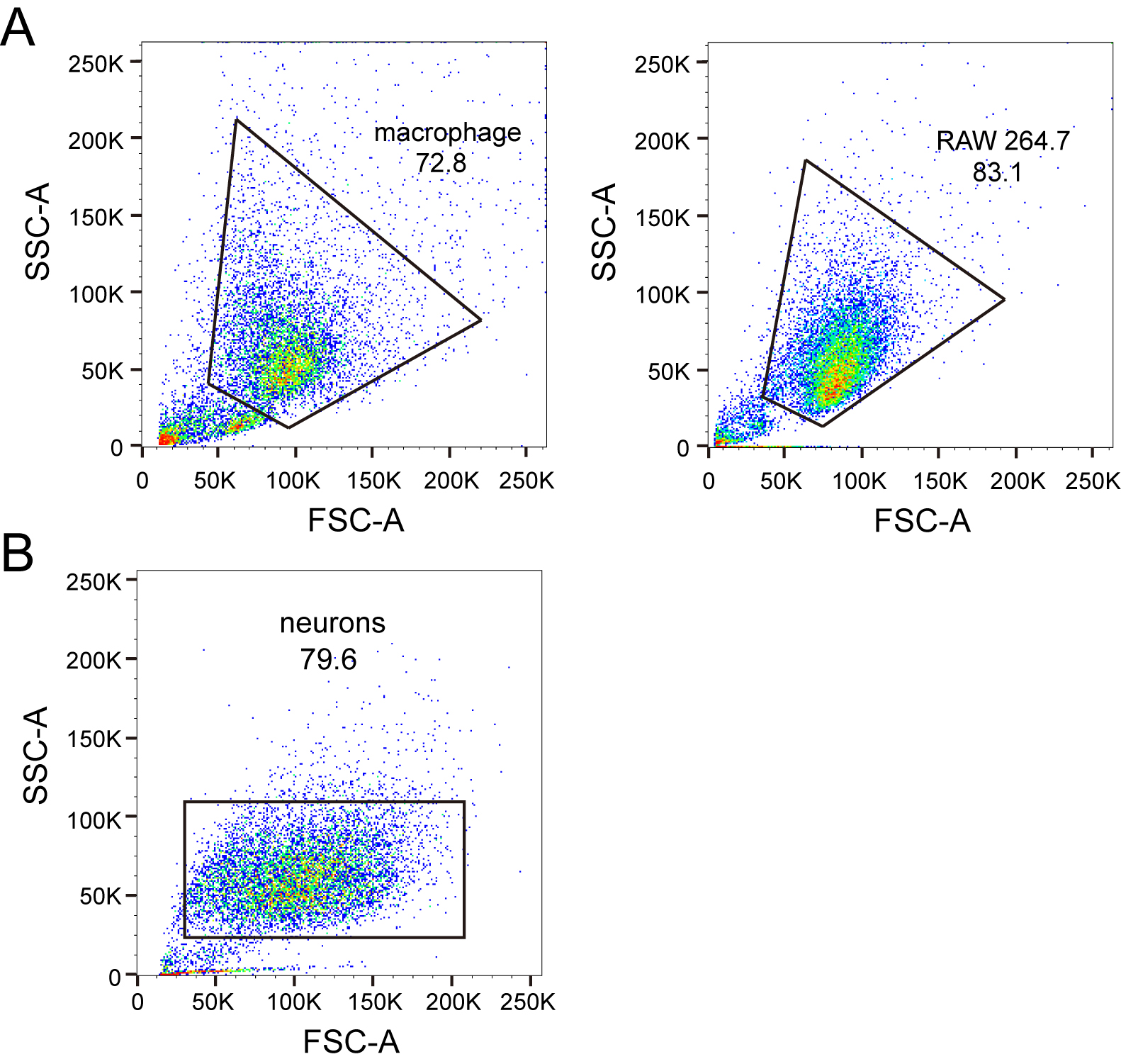

Supplement: Supplementary file 3 — Additional file 3 : Figure S2. (a) Flow cytometry plots showing the gating strategy to identify macrophages and RAW264.7 cells. SSC-A = side scatter-area; FSC-A = forward scatter-area. (b) Flow cytometry plots showing the gating strategy to identify neurons. SSC-A = side scatter-area; FSC-A = forward scatter-area. [file 12974_2020_1735_MOESM3_ESM.jpg]

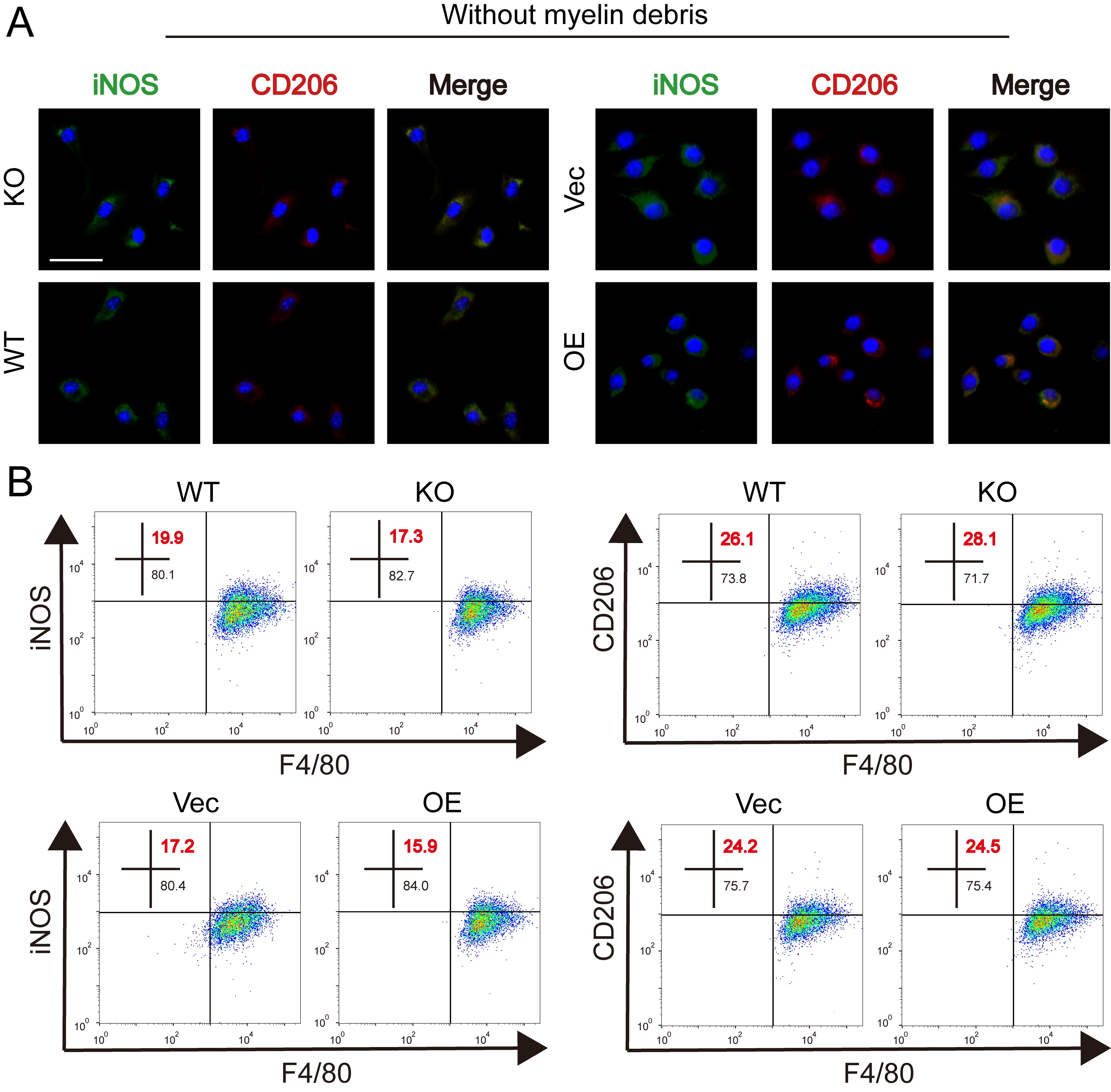

Supplement: Supplementary file 4 — Additional file 4 : Figure S3. (a) IF staining of iNOS, CD206, and F4/80 in different groups of macrophages and RAW264.7 cells (WT vs KO, Vec vs OE) in absence of myelin debris, n=3 per group. Scale bar = 20 μm. (b) Flow cytometric analysis of iNOS, CD206, and F4/80 in different groups of macrophages and RAW264.7 cells (WT vs KO, Vec vs OE) in absence of myelin debris, n=3 per group. [file 12974_2020_1735_MOESM4_ESM.jpg]

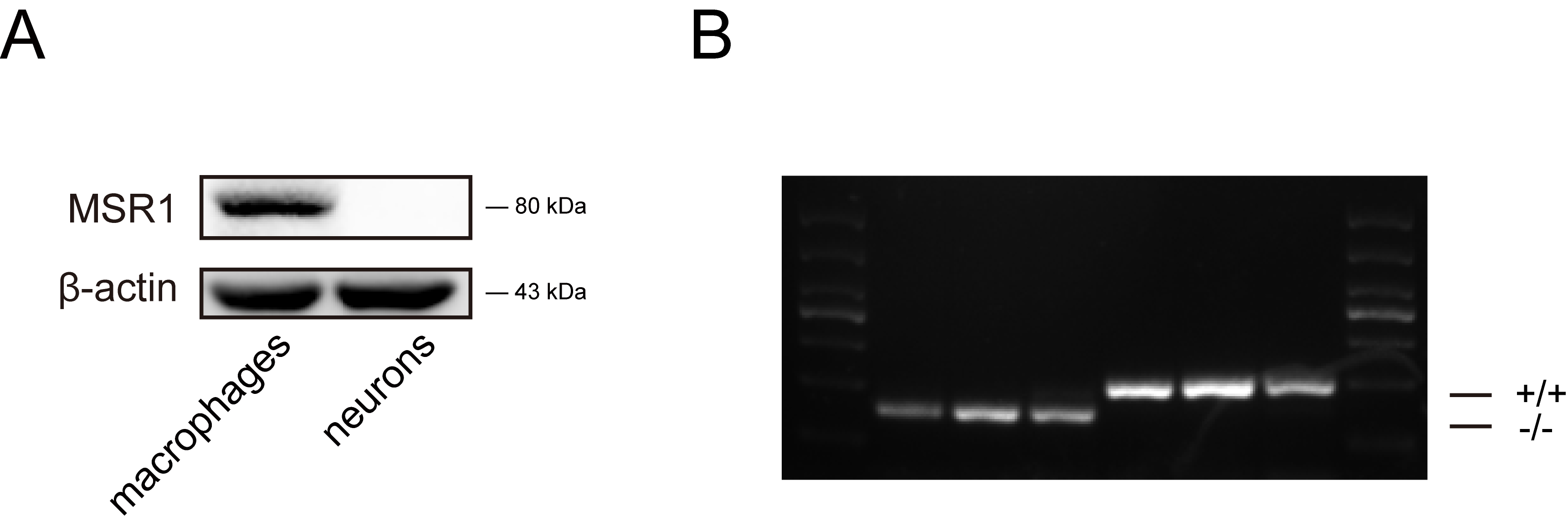

Supplement: Supplementary file 5 — Additional file 5 : Figure S4. (a) Immunoblot images showing the expression patterns of MSR1 in macrophages and neurons, n=3 per group. (b) The genotyping of MSR1 WT or MSR1 KO mice was confirmed by PCR of DNA samples from tail chips. [file 12974_2020_1735_MOESM5_ESM.jpg]
